# Cognitive and biological effects of citrus phytochemicals in subjective cognitive decline: a 36-week, randomized, placebo-controlled trial

**DOI:** 10.1186/s12937-022-00817-6

**Published:** 2022-10-17

**Authors:** Samantha Galluzzi, Roberta Zanardini, Clarissa Ferrari, Sara Gipponi, Ilaria Passeggia, Michela Rampini, Giovanni Sgrò, Salvatore Genovese, Serena Fiorito, Lucia Palumbo, Michela Pievani, Giovanni B. Frisoni, Francesco Epifano

**Affiliations:** 1 Laboratory Alzheimer’s Neuroimaging and Epidemiology, IRCCS Istituto Centro San Giovanni Di Dio Fatebenefratelli, Brescia, Italy; 2 Molecular Markers Laboratory, IRCCS Istituto Centro San Giovanni Di Dio Fatebenefratelli, Brescia, Italy; 3 Service of Statistics, IRCCS Istituto Centro San Giovanni Di Dio Fatebenefratelli, Brescia, Italy; 4 Clinical Trial Service, IRCCS Istituto Centro San Giovanni Di Dio Fatebenefratelli, Brescia, Italy; 5 Laboratory of Phytochemistry and Chemistry of Natural Products, Department of Pharmacy, University “G. d’Annunzio” of Chieti-Pescara, Chieti, Italy; 6 University Hospitals and University of Geneva, Geneva, Switzerland

**Keywords:** Subjective cognitive decline, Randomized clinical trial, Auraptene, Naringenin, Biological markers

## Abstract

**Background:**

Auraptene (AUR) and naringenin (NAR) are citrus-derived phytochemicals that influence several biological mechanisms associated with cognitive decline, including neuronal damage, oxidative stress and inflammation. Clinical evidence of the efficacy of a nutraceutical with the potential to enhance cognitive function in cohorts at risk of cognitive decline would be of great value from a preventive perspective. The primary aim of this study is to determine the cognitive effects of a 36-week treatment with citrus peel extract standardized in levels of AUR and NAR in older adults experiencing subjective cognitive decline (SCD). The secondary aim is to determine the effects of these phytochemicals on blood-based biomarkers indicative of neuronal damage, oxidative stress, and inflammation.

**Methods:**

Eighty older persons with SCD will be recruited and randomly assigned to receive the active treatment (400 mg of citrus peel extract containing 0.1 mg of AUR and 3 mg of NAR) or the placebo at a 1:1 ratio for 36 weeks. The primary endpoint is a change in the Repeatable Battery for the Assessment of Neuropsychological Status score from baseline to weeks 18 and 36. Other cognitive outcomes will include changes in verbal and nonverbal memory, attention, executive and visuospatial functions. Blood samples will be collected from a consecutive subsample of 60 participants. The secondary endpoint is a change in interleukin-8 levels over the 36-week period. Other biological outcomes include changes in markers of neuronal damage, oxidative stress, and pro- and anti-inflammatory cytokines.

**Conclusion:**

This study will evaluate whether an intervention with citrus peel extract standardized in levels of AUR and NAR has cognitive and biological effects in older adults with SCD, facilitating the establishment of nutrition intervention in people at risk of cognitive decline.

**Trial registration:**

The trial is registered with the United States National Library of Medicine at the National Institutes of Health Registry of Clinical Trials under the code NCT04744922 on February 9^th^, 2021 (https://www.clinicaltrials.gov/ct2/show/NCT04744922).

## Background

Alzheimer’s disease (AD) is the fifth leading cause of death among those aged 65 and older and is also a leading cause of disability and poor health [1]. The economic burden of treating patients is overwhelming, and is estimated to increase in the coming years as the population ages [2]. Simulation studies have suggested that a focus on treatments that provide even short delays in onset of dementia will have immediate impacts on longevity, quality of life, and reduced incidence of dementia [3].

Subjective cognitive decline (SCD) in older adults refers to an individual’s subjective perception of a decline in cognitive function in the absence of objective evidence [4]. Longitudinal studies on SCD have shown that the annual incidence rates of progression to mild cognitive impairment were between 5 and 16%, depending on the study setting [5]. SCD has also been characterized by pathological changes in the brain associated with AD [6]. These findings suggest that SCD may be an early-stage marker for AD and a potential target to test interventions aimed at maintaining cognitive function as long as possible. Thus, high-quality studies in this field are warranted [7].

Accumulating evidence suggests that a nutritional diet based on fruits and vegetables is important for optimizing cognition and reducing the risks of dementia and AD [8, 9]. Dietary interventions with plant-derived nutraceuticals are an attractive approach to enhance cognition, in light of their relatively low cost relative to synthetic substances; low adverse effects profile, which results in increased compliance [10]; and their effects on numerous brain systems associated with cognitive decline [11]. Experimental data indicate that nutraceuticals benefit cognitive function [12, 13, 14, 15], but clinical data from controlled interventions in elderly individuals are still preliminary.

Naringenin (NAR) and auraptene (AUR) are citrus-derived phytochemicals categorized as polyphenols and oxyprenylated secondary metabolites, respectively, that belong to the flavonoid and coumarin classes. Preclinical studies have shown that they exert anti-inflammatory, antioxidant, and neuroprotective effects in mouse models of brain damage [16, 17, 18, 19, 20, 21, 22] and, specifically, in mouse models of AD [23, 24, 25, 26, 27]. In particular, NAR improved spatial learning and memory performance in ageing mice through a reduction in a-beta production, tau hyperphosphorylation, oxidative stress, and neuroinflammation in the brain [27]. Auraptene markedly reversed impairments in the retention of avoidance memory that were induced by scopolamine in mice [24] and showed neuroprotective and anti-inflammatory properties [19]. Moreover, the combined administration of AUR and NAR suppressed neuronal cell death in the hippocampus by inhibiting neuroinflammation [28].

While experimental research provides a strong preclinical rationale for the use of AUR and NAR to enhance cognition, clinical studies supporting their beneficial effect on cognition in humans are sparse. Studies of polyphenol-rich fruit juices, including those from citrus species, have suggested positive effects on cognition in older adults, namely, global cognition and verbal memory [12]. However, the lack of an accurate chemical definition of the plant-derived products makes it difficult to assess the contribution of a single phytochemical. To the best of our knowledge, only one randomized, placebo-controlled, double-blind study has evaluated the cognitive effect of AUR administration in older adults; this study showed significantly greater improvements in immediate memory in the AUR group than in the placebo group [29]. The few clinical trials that have evaluated the clinical effects of NAR focused on cardiovascular risk factors, not cognition [30].

The primary aim of this trial is to determine the cognitive effect of a 36-week treatment with citrus peels extract standardized in levels of AUR and NAR on older adults with SCD. The secondary aim is to determine the effect of this treatment on blood-based biomarkers indicative of neuronal damage, oxidative stress, and inflammation. We defined SCD according to international research criteria [31] using a semistructured interview [32]. In addition, we will administer two self-report questionnaires, which we chose on the basis of psychometric properties and the availability of Italian versions to assess SCD.

## Methods

### Trial design and setting

The study is a randomized, double-blind, placebo-controlled, 36-week trial to evaluate the cognitive and biological effects of citrus peel extract standardized in levels of AUR e NAR on 80 older adults with SCD. The current study protocol was designed in accordance with the consolidated standards of reporting trials (CONSORT) [33] and the recommendations of the International Academy on Nutrition and Aging Task Force [34]. The study is being conducted at the Laboratory of Alzheimer’s Neuroimaging and Epidemiology (LANE) of the IRCCS Istituto Centro San Giovanni di Dio Fatebenefratelli, Brescia, Italy.

Participants will be recruited by several methods, including advertisements in local newspapers and internet newsletters, flyers located at our Institute, the internet (via the LANE [35] and Institute websites [36]), and social media promotion through Facebook. In addition, participants will be contacted from a list of people who took part in previous research studies conducted at the LANE and indicated they were available for future research. Retention will be facilitated by regular contact during the clinical trial for screening, appointment reminders, and the testing sessions.

Recruitment of participants started in April 2021 and is expected to run until April 2022. The last follow-up is scheduled for February 2023.

### Ethics approval

The protocol (version 3.0) was approved by the local ethical committee, the Ethics Committee of the IRCCS Istituto Centro San Giovanni di Dio Fatebenefratelli. Any modification to the study objectives, study design, participant population, sample size, study procedures, or significant administrative aspects will require an amendment to the protocol. All participants must provide informed verbal consent via telephone and written informed consent at the time of face-to-face screening to a member of the research team. The research will be conducted in accordance with the International Conference on Harmonization of Good Clinical Practice (GCP/ICH) guidelines and was performed in line with the principles of the Declaration of Helsinki.

### Inclusion and exclusion criteria

The inclusion criteria are as follows: (i) subjects between 60 and 75 years old and (ii) those who exhibit SCD according to research criteria proposed by the SCD-I working group [31], and (iii) subjects who perform within the normal range on standardized cognitive tests (scores are corrected for age and education, according to Italian normative populations). The SCD criteria were operationalized to include features of the SCD *plus* category, specifically the presence of a subjective decline in memory, rather than other domains of cognition, onset of SCD within the last 5 years, and worries associated with SCD expressed by the participant and/or an informant;

The exclusion criteria are as follows: (i) cognitive performance below the normal range on two tests within a single cognitive domain (i.e., memory, executive function, or attention); (ii) the presence of current major neurological (including stroke, dementia or cognitive impairment, and cancer) or psychiatric (including major depressive disorder, bipolar disorder, and drug and alcohol dependence) disorders, according to the International Statistical Classification of Diseases and Related Health Problem, 10^th^ revision and/or Diagnostic and Statistical Manual of Mental Disorders, 5^th^ edition criteria; (iii) severe depressive symptoms, as indicated by scores > 17 on the 30-item Geriatric Depression Scale [37] (current psychotropic therapy allowed if at a stable dose over the previous 8 weeks); (iv) the presence of a chronic disease or acute unstable illness (respiratory, cardiovascular, digestive, renal, metabolic, haematologic, endocrine, infectious, or malignant) that would interfere with the aims of the study protocol; and (v) the use of supplements that could interfere with the study nutraceutical (e.g. cognitive enhancers). Current use of supplements is allowed if at a stable dose over the previous 8 weeks and maintained at a constant dose for the duration of the study.

### Study schedule

**Fig. 1:**
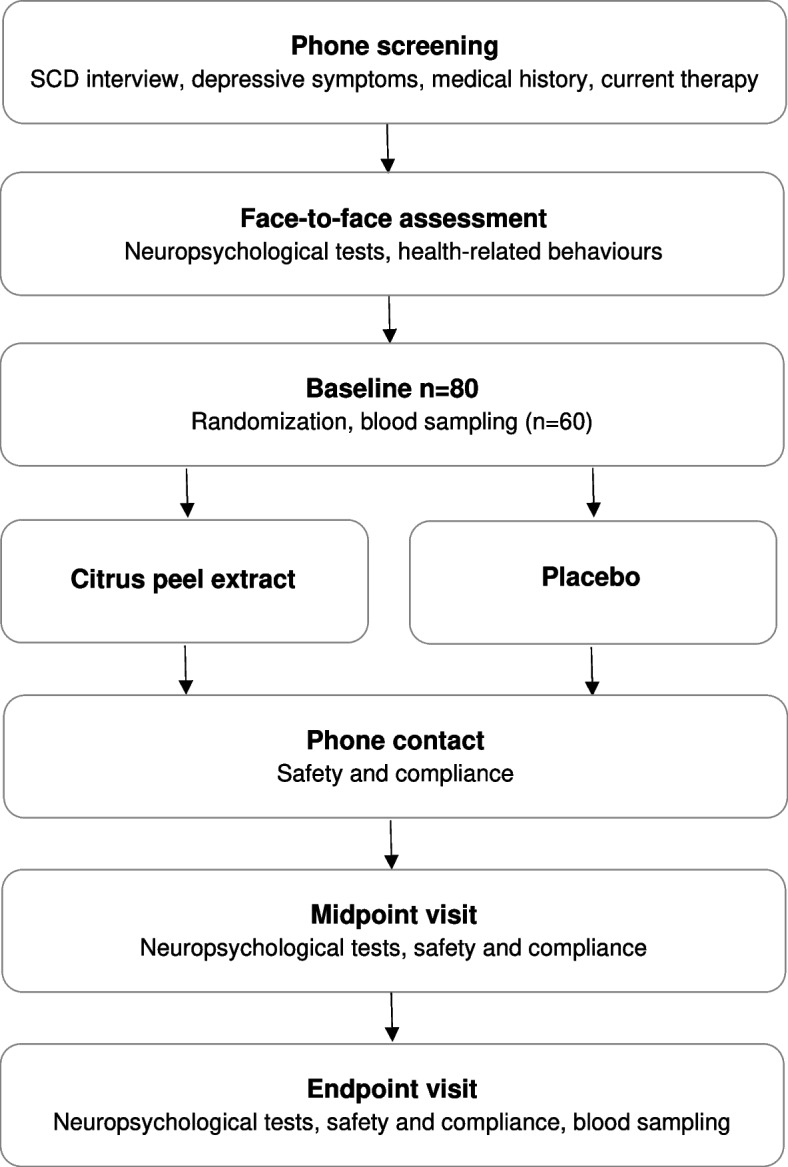
Flowchart of the trial

The study schedule of the trial protocol is detailed in the Fig. 1. Phone screening will be conducted with participants to determine their preliminary eligibility with regards to the above inclusion and exclusion criteria. The Subjective Cognitive Decline-Interview [32] and 30-item Geriatric Depression Scale [37] will then be administered and data on medical history and current medications will be collected. The Cumulative Illness Rating Scale [38] will also be completed.

Upon a successful phone screening, participants will be invited to undergo a face-to-face assessment and cognitive battery (see the paragraph below). In addition, data on a number of health-related behaviours will be collected. Subjects will be asked about their alcohol consumption (current and past numbers of drinks/day in terms of wine, beer, liquor), tobacco use (current and past numbers of cigarettes/day), tea consumption (current and past numbers of cups/day), and lemon consumption (number and frequency). The 14-point Mediterranean Diet Adherence Screener [39] will be administered to evaluate the Mediterranean diet adherence, and the Cognitive Reserve Index questionnaire [40] will be administered to measure cognitive reserve. Symptoms of anxiety will be evaluated with the State-Trait Anxiety Inventory [41].

Eligible participants will then be invited to the baseline visit, which was scheduled within 7 days of the face-to-face assessment, and allocated to one of the two intervention groups. A blood sample will be collected for biomarker measurement in a subsample of 60 consecutive subjects. Follow-up visits will include (i) a phone call after 4 weeks (± 3 days) from the baseline to assess compliance and safety; (ii) a midpoint visit 18 weeks (± 1 week) after baseline to assess cognition, compliance and safety; and (iii) an endpoint visit 36 weeks (± 1 week) after baseline to assess cognition, compliance, safety, and to collect a second round of blood samples for biomarker measurement.

### Randomization

Participants will be randomly allocated at a 1:1 ratio to either the treatment or placebo group. A blockwise (block size of 6) randomization sequence was generated using a computer-based algorithm from a statistician not directly involved in the recruitment or assessment of participants. The block size of 6 was due to logistical procedures of the trial (planned enrolment of six subjects per month). The sequence was obtained by a random generator of 6 labels (‘T’ for treatment or ‘P’ for placebo) in which the categories T or P had the same probability of occurring. Opaque, sealed envelopes will be used to conceal the sequence until the intervention is assigned. This is a double-blind trial, so both the participants and research team will remain blinded to treatment allocation until study completion. Unblinding is permissible in cases of medical emergencies or serious medical conditions that occur while a participant takes part in the study.

### Compliance and adverse effects

Compliance will be assessed by instructing participants to return any unused medication at the midpoint and endpoint. A diary for noting medication intake and adverse effects will be provided to the participants, with instructions to record when new medications were taken or adverse events occurred.

We expect this trial to have minimal risks to participants. Adverse events will be closely monitored throughout the course of the study. In particular, participants will be instructed to immediately contact the research team in case of unexpected medical care visits or serious adverse events/hospitalization. Any adverse events will be reported to the Ethics Committee of the IRCCS Istituto Centro San Giovanni di Dio Fatebenefratelli. The outcome and actions taken will be recorded. If a serious or unexpected adverse event related to trial procedures occurs, participants will have provisional care beyond that immediately needed.

In the case of discontinuation, the subject will be retained in the trial whenever possible to enable follow-up data collection and prevent missing data. Reasons for discontinuation will be recorded.

### Primary and secondary endpoints

The primary endpoint is the change in the Repeatable Battery for the Assessment of Neuropsychological Status (R-BANS) score from baseline to weeks 18 and 36. The secondary endpoint is the change in interleukin-8 levels over the 36-week trial period.

### Intervention

The trial nutraceutical manufacturer is the Laboratory of Phytochemistry and Chemistry of Natural Products, Department of Pharmacy, University ‘G. d’Annunzio’ of Chieti-Pescara, Chieti, Italy.

*Active*. The active treatment consists of one capsule a day (morning) containing 400 mg of *Citrus limon* (L.) Osbeck (Fam. Rutaceae) (common name “lemon”) peel extract standardized in levels of AUR e NAR along with 400 mg of starch (the same inert substance used as the placebo) for 36 weeks. The starch was added to facilitate filling the capsules to the maximum capacity. The capsules were stored at room temperature.

The plant material came from crops without any chemical and/or phytosanitary treatment (e.g. pesticides) planted in lands owned by one of the research teams in Barcellona Pozzo di Gotto (Sicily region, Italy) and was identified from a taxonomic perspective by authors from Chieti. Fresh peels were first homogenized by an Ultra Turrax® apparatus and the semisolid material was liophylized to obtain a fine powder. No solvents were used to obtain this dry extract. A voucher specimen (named LPENat-2021) of this powder is stored in the repository of the Laboratory. The composition of the trial nutraceutical components according to the reference compounds is shown in the Table 1. The daily dose is 0.1 mg of AUR and 3 mg of NAR.

**Table 1:** Auraptene, naringenin, and total flavonoid and polyphenolic composition of the trial nutraceutical (concentration expressed as mg/g of dry powder ± S.D)

Auraptene	0.223 ± 0.002
Naringenin	7.472 ± 0.008
Total flavonoids	148.815 ± 0.021
Total polyphenoles	302.442 ± 0.032

The HPLC analyses for the quantification of AUR and NAR were carried out following the guidelines provided by The International Council for Harmonization of Technical Requirements for Pharmaceuticals for Human Use (ICH) [42]. The determination of total flavonoids and total polyphenols was accomplished following standard procedures [43]. Other purity testing included heavy metals (performed by inductively coupled plasma analysis) providing data that they were largely within the limits set by the current reference legislation (USP 232, 233 and ICQ3D) [44].

*Placebo*. The placebo capsule contained an inert substance (800 mg of starch), matched for colour and smell to that of the active treatment. To achieve this match, the outer surface of the containers provided to the placebo group was aerosolized with an ethanolic solution of the lemon peel powder used in the treatment group. The dosage was one capsule a day (morning) for 36 weeks.

### Data collection

*Cognitive battery.* A number of standardized neuropsychological tests were selected for this trial.

The primary endpoint of the study is a change in the total index score on the R-BANS [45] from baseline to weeks 18 and 36. This neuropsychological test battery includes 12 standard cognitive subtests, which yield an age-adjusted total index score and five age-adjusted index scores for the following cognitive domains: immediate memory (list learning and story memory), visuospatial/constructional (figure copy and line orientation), language (picture naming and semantic fluency), attention (digit span and digit symbol coding) and delayed memory (list recall, list recognition, story recall and figure recall). The total score ranges from 40 to 160. Two Italian-validated forms will be used (Forms A and B) to prevent a learning effect from serial assessments.

Other cognitive outcomes include the mean change in global cognition (Mini Mental State examination [46]), verbal memory (California Verbal Learning test [47]), attention (Attentional Matrices [48]; Stroop test [49]; Trail Making Test A [50]), executive functions (Trail Making Test B [50]; Wisconsin Card Sorting test [51]), visuospatial functions (Clock Drawing test [52]), and scales of memory concerns (Everyday Memory Questionnaire [53]; Multifactorial Memory Questionnaire [54]) (Table 2).

**Table 2:** Cognitive outcomes measured in the trial

*Cognitive domain*	*Cognitive test*
*Global cognition*	*R-BANS [45]
	Mini mental state examination [46]
*Verbal memory*	California verbal learning test [47]
*Attention and executive functions*	Attentional matrices [48]
	Stroop test [49]
	Trail making test [50]
	Wisconsin card sorting test [51]
*Visuospatial functions*	Clock drawing test [52]
*Memory concerns*	Everyday memory questionnaire [53]
	Multifactorial memory questionnaire [54]

*R-BANS* Repeatable Battery for the Assessment of Neuropsychological Status

^*^Primary endpoint

*Biological measures.* Fasting venous blood samples will be collected at the baseline and the endpoint using EDTA-coated and anticoagulant-free tubes. Samples will be kept at 4 °C for 20 min to 1 h until centrifugation for 15 min (4 °C, 1000 × g). Plasma will be collected and centrifuged 5 min (4 °C, 1,000 × g) after the addition of the 1X protease inhibitors. Finally, plasma and serum will be aliquoted and stored at –80 °C until analysis.

The secondary endpoint of the study is the change in interleukin-8 (IL-8) levels over the 36-week trial period. These levels will be measured by a multiplex immunoassay system using magnetic beads.

Other biological outcomes include a panel of markers indicative of neuronal damage, oxidative stress and inflammation, measured by different techniques (Table 3).

**Table 3:** Blood-based biological outcomes measured in the trial

*Biological marker*	*Method of measurement*
**Neuronal damage**	
BDNF, IGF-1	ELISA
NF-light	Simoa SR-X, quanterix
**Oxidative stress**	
Nitric oxide, thiobarbituric acid reactive substances, superoxide dismutase	ELISA
**Inflammation**	
*Pro-inflammatory cytokines*	
*CXCL8/IL-8, IL-1beta, IL-6, IL-17, TNF-alpha, IP10, MIP-1alfa, CCL2/MCP-1, CCL5/RANTES, IL-18	Bio-plex 200, Bio-Rad
*Anti-inflammatory molecules*	
IL-10, IL1-Ra, soluble TNF receptor I, soluble TNF receptor II	Bio-plex 200, Bio-Rad

*BDNF* Brain-Derived Neurotrophic Factor, *IGF* Insulin-like Growth Factor, *NF* NeuroFilament, *CXCL8* C-X-C motif Chemokine Ligand 8, *IL* InterLeukin, *TNF* Tumour Necrosis Factor, *IP* Interferon γ-induced Protein, *MIP* Macrophage Inflammatory Protein, *CCL2/MCP-1* C–C motif Chemokine Ligand 2/Monocyte Chemoattractant Protein-1, *CCL5/RANTES* C–C motif Chemokine Ligand 5/ Regulated on Activation Normal T cell Expressed and Secreted, *Ra* Receptor antagonist

^*^Secondary endpoint

### Data management

Data collection forms and protocols will be accessible to study investigators in a secured shared drive. Data collected at the visits will be entered electronically by the study coordinators (IP and MR) and checked by the principal investigator (SGa). Only study investigators will be able to access the password-protected participant data and all information will remain confidential. To ensure confidentiality, data disseminated to other study investigators will be deidentified. Participant files will be locked and secured outside working hours.

To ensure confidentiality, participants will be identified by a coded identification number. This coded identification number will be used to identify all laboratory specimens, data collection, and administrative forms to maintain participant confidentiality.

The study coordinators and principal investigator will have access to the final trial dataset. The principal investigator will oversee intrastudy data sharing. To ensure confidentiality, data shared among other study investigators will be deidentified.

### Data monitoring

The study coordinators will oversee all aspects of data monitoring with the assistance of the principal investigator. Due to the scale and short duration of this trial, a data monitoring committee is not needed. We also expect participants to experience minimal risks when undertaking this trial; therefore, no interim analyses will be conducted.

### Statistical analysis

*Sample size.* For the primary endpoint, we computed an appropriate sample size based on an interventional study [55] in older adults with SCD that measured the same cognitive outcome as our study. In that study, a change of 6.8 points in the R-BANS score was observed in the treatment group (baseline mean = 95.9, standard deviation SD = 11.1; posttreatment mean = 102.7, SD = 9.9). To compute the sample size of our study, the following hypotheses were made: i) an SD at baseline equal to 11 in both study groups (treatment and placebo); ii) a correlation between baseline-post treatment of the R-BANS values of 0.7, corresponding to an SD of about 8.5; iii) a change in the placebo group (precautionary hypothesis) of 20% with respect to the change in treatment group (i.e., a change of 1.4 points in the R-BANS score in the placebo arm). Under these hypotheses, by using a two-tailed t test with a significance level alpha = 0.05 and a power of 0.8, the minimum appropriate sample size for detecting a significant difference between the pre-post changes in the two groups was *N* = 80 (40 placebo vs. 40 treated).

For the secondary endpoint, we computed an appropriate sample size based on a study by Sanchez-Rodriguez et al. [56] who measured the biological effect of a nutraceutical in heathy adults. In that study, a mean pre-post difference of 0.2 points was found in IL-8 levels (mean: 1.6 pg/mL (SD = 0.8) vs 1.4 pg/mL (SD = 0.8)). As the duration of the intervention in our study will be longer (36 vs. 3 weeks), we assumed a mean change twice as large as that in their study (0.4 points). Given a two-tailed nonparametric Wilcoxon rank test for matched pairs, an alpha level = 0.05, power = 0.8 and a pre-post correlation of 0.8, an effect size of 0.65 was found. To detect a significant result with a similar effect size, a sample size of *n* = 22 subjects per group (22 treated vs. 22 untreated) was necessary. We therefore set the sample size to *n* = 60 (30 per arm) considering it a good trade-off between cost feasibility and statistical power.

*Data analysis.* Descriptive statistics on cognitive and biological markers in the two groups of treatment will be presented as the mean and standard deviation or the median and quartiles. Longitudinal comparisons will be performed using the intention-to-treat approach with linear and/or generalized linear mixed models. In particular, different models will be carried out with cognitive scores and biological markers as the dependent variables and time (baseline and follow-up) as the within-subject factor. The per-protocol analysis will be carried out with individuals who complete over 80% of each assigned condition.

*Dissemination*. The results from the trial will be publicly presented to scientific researchers and health care professionals through peer-reviewed journals and scientific conference presentations. The authorship requirements will adhere to scientific journal guidelines. The results will also be made available for scientific and lay audiences on the ClinicalTrials.gov website. In addition, individual trial outcomes will be forwarded to participants, with their consent. We will transmit findings, when appropriate, to the general population using media coverage, such as newspaper articles and television interviews.

## Discussion

This article presents the rationale and methods for a pilot clinical trial on the cognitive and biological effects of citrus peel extract standardized in levels of AUR and NAR on older adults with SCD. Currently, limited treatments are available for elderly individuals reporting SCD who are potentially at risk of developing cognitive impairment and dementia. Therefore, it is important to test the efficacy of interventions that may promote cognitive health and ultimately prevent cognitive decline [7].

The nutraceuticals have shown promise in cognitive enhancement, but only a few randomized clinical trials have evaluated their effect on SCD. In a recent systematic review of the effects of nutrition on cognitive function in healthy adults, only 6 out of 48 interventions with dietary supplements were carried out on subjects with SCD [57]. Of these 6 studies, only one evaluated the effect of a long-term (24 weeks) intervention in older adults with SCD defined according to the international criteria [31]. In half of these 6 studies, concerns have been raised about quality. Last, although antioxidant and anti-inflammatory properties have been recognized as the potential mechanisms linking dietary supplements with cognitive functions, none of six studies included biological biomarkers as surrogate outcomes of efficacy. Our pilot clinical trial overcame these limitations and will provide preliminary evidence in the emerging field of using dietary interventions for SCD.

The primary endpoint of the trial is the R-BANS total score. The R-BANS is a well-established clinical tool that was specifically designed for diagnosis, tracking, and clinical trial outcome measurement in early and prodromal AD [58]. Additionally, the R-BANS is one of the cognitive measures recommended for drug trials and research on preclinical AD [59]. The other cognitive outcome measures included in the trial were cognitive tests sensitive to ageing and early dementia. In particular, the California Verbal Learning, Trail Making, and Clock Drawing tests have been used in AD prevention trials [60]. The Stroop test, attentional matrices, and Wisconsin Card Sorting test have been administered in studies of nondemented older adults [61, 62] and SCD [63].

Regarding the self-reported SCD measures, consensus about which questionnaires should be used is lacking [64]. We decided to adopt the Everyday Memory Questionnaire because we found it to be a reliable indicator of subjective memory complaints in older adults [53] and in subjects with SCD [65]. The other questionnaire, Multifactorial Memory, was an Italian version with robust psychometric properties, ensuring comparability among studies in different countries. Moreover, it specifically taps metamemory, i.e., knowledge about one’s own memory, which is a sensitive measure of SCD intervention efficacy [7],

The secondary endpoint of the trial is the change in IL-8 levels. The IL-8 is a proinflammatory cytokine that can contribute to the pathophysiology of AD [66, 67]. A study showed that elevated IL-8 levels were associated with worse memory and cognitive speed in healthy elderly individuals [68], suggesting its potential role as a surrogate biological marker of early cognitive dysfunction. As experimental studies have shown that AUR and NAR decrease IL-8 levels [69, 70], we believed that levels of this cytokine were an appropriate method of evaluating the biological effect of citrus phytochemicals in cognitively frail elderly individuals. The other biological outcome measures were related to biological pathways linking dietary interventions with cognitive function improvement. Due to financial constraints, we selected a small, but representative panel of biomarkers indicative of pathways that benefit from AUR and NAR. They included levels of brain-derived neurotrophic factor and insulin-like growth factor-1 [71, 72], nitric oxide, thiobarbituric acid reactive substances, and superoxide dismutase [71, 73], and pro- and anti-inflammatory molecules and cytokines [74, 75]. In addition, we included neurofilament light chain because this protein is becoming increasingly valuable as a peripheral biomarker of neuronal damage and subjective cognitive decline [76].

We used citrus peels as the source of the nutraceutical administered in the trial because they are among the richest natural sources of AUR and NAR [77, 78]. Thanks to favourable climatic and geographical conditions in southern Italy—the region from which the lemons used in this study originated—*Citrus* plants are easily grown. This would allow for large-scale in-house production of the nutraceutical under study. As multiple phytochemicals work synergistically with each other to produce cognitive benefits [12], we decided to manufacture the nutraceutical using the whole citrus peel extract instead of the individual phytochemicals. However, we conducted a complete chemical characterization of the whole extract to allow result comparison of our results with those of other studies.

We established the trial nutraceutical dosage based on estimated data of flavonoid intake and major food sources of the elderly individuals [79]. Even though these estimations may be subject to methodological limitations, such as overlooking individual bioavailability and metabolism in the human body, they provide a basis to study the effect of dietary supplementation in reducing the risks of chronic diseases in healthy people [80]. In particular, our trial nutraceutical provided approximately 60 mg/day of total flavonoids, which is equal to or greater then the flavonoid intake estimated in elderly individuals according to analysis of food samples or food tables [79]. The selection of a placebo-only control group as a comparator was justified because no interventions are available for SCD.

The health risks of the studied citrus phytochemicals are expected to be minimal. No side effects of AUR administration in animal models [81] or in older adults [29] have been reported. A recent randomized clinical trial that administered NAR to healthy adults showed no relevant adverse events or changes in blood safety markers [82]. Possible side effects of citrus phytochemicals will be monitored.

We designed the trial following the recommendations of the International Academy on Nutrition and Ageing Task Force for trials of nutritional interventions to slow cognitive decline in older adults [34]. Accordingly, our target population was represented by frail elderly individuals, i.e., subjects with SCD, who are at increased risk of cognitive decline. Although SCD alone is not sufficient to identify preclinical AD in the absence of AD biomarkers, we included older adults with characteristics of SCD *plus*, which are associated with an increased likelihood of underlying AD pathology [83]. Second, the length of the trial was 36 weeks, which is a longer treatment period than that of other studies of nutraceuticals for SCD. As small cognitive effects have been shown in trials lasting from 8 to 24 weeks [84, 85, 86, 87, 88], we expect that a longer trial period, including midpoint testing, will provide more information on the trajectory of cognitive changes. Last, the inclusion of biological markers will provide surrogate measures of treatment efficacy and allow us to identify possible mechanisms of action underlying the proposed treatment-associated benefits on cognition, as recommended for AD drug development [89].

### Limitations

A limitation of the study is the lack of genetic (apolipoprotein E, *APOE* gene) and biological (AD biomarkers) characterizations in our trial population. In particular, the *APOE4* allele is the major genetic risk factor for AD and can influence the rate of cognitive decline in SCD subjects [90]. The exploratory nature of our study prevented equal allocation of participants into study groups according to the *APOE4* allele and the provision of a biological AD signature of the participants.

## Conclusions

Positive results from this study will inform the design of larger clinical trials of longer duration to test citrus phytochemicals supplementation as a strategy for AD prevention and will allow the public health organizations to plan interventions for at-risk older individuals.

## Data Availability

Not applicable.

## Abbreviations

AD: Alzheimer’s disease
SCD: Subjective cognitive decline
NAR: Naringenin
AUR: Auraptene
LANE: Lab Alzheimer’s Neuroimaging and Epidemiology
R-BANS: Repeatable Battery for the Assessment of Neuropsychological Status
IL-8: interleukin-8
*APOE*: apolipoprotein E
